# Necrosis targeted combinational theragnostic approach to treat cancer

**DOI:** 10.18632/oncotarget.1728

**Published:** 2014-03-11

**Authors:** Yun Ji, Cuihua Jiang, Xueli Zhang, Wei Liu, Meng Gao, Yue Li, Junhu Wang, Qingqing Wang, Ziping Sun, Xiao Jiang, Nan Yao, Xiaoning Wang, Zhijun Fang, Zhiqi Yin, Yicheng Ni, Jian Zhang

**Affiliations:** ^1^ Laboratory of Translational Medicine, Jiangsu Province Academy of Traditional Chinese Medicine, Nanjing 210028, Jiangsu Province, P.R.China; ^2^ Department of Natural Medicinal Chemistry & State Key Laboratory of Natural Medicines, China Pharmaceutical University, Nanjing 210009, Jiangsu Province, P.R.China; ^3^ Department of Nuclear Medicine, The First Affiliated Hospital of Nanjing Medical University, Nanjing 210029, Jiangsu Province, P.R.China; ^4^ Radiation Medical Institute, Shandong Academy of Medical Sciences, Jinan 250062, Shandong Province, P.R.China; ^5^ Department of Radiology, Faculty of Medicine, K.U. Leuven, BE 3000, Leuven, Belgium

**Keywords:** 131-iodine radioiodinated sennidin A (^131^I-SA), rodent tumor models, combretastatin A4 phosphate (CA4P), diagnosis, necrosis targeting radiotherapy

## Abstract

Residual cancer cells and subsequent tumor relapse is an obstacle for curative cancer treatment. Tumor necrosis therapy (TNT) has recently been developed to cause residual tumor regression or destruction. Here, we exploited the avidity of the sennidin A (SA) tracer and radioiodinated SA (^131^I-SA) to necrotic tumors in order to further empower TNT. We showed high uptake and prolonged retention of SA in necrotic tumors and a quick clearance in other non-targeted tissues including the liver. On SPECT-CT images, tumor mass appeared persistently as a hotspot. Based on the prominent targetability of ^131^I-SA to the tumor necrosis, we designed a combinational theragnostic modality. The vascular disrupting agent (VDA) combretastatin A4 phosphate (CA4P) was used to cause massive tumor necrosis, which formed the target of ^131^I-SA that subsequently killed the residual tumor cells by cross-fire irradiation of beta particles. Consequently, ^131^I-SA combined with CA4P significantly inhibited tumor growth, extended tumor doubling time and prolonged mean animal survival. In conclusion, ^131^I-SA in combination with necrosis inducing drugs/therapies may generate synergetic tumoricidal effects on solid malignancies by means of primary debulking and secondary cleansing process.

## INTRODUCTION

Cancer remains a major cause of human suffering and death worldwide. New developments of radiotherapy and chemotherapy have made the progress in cancer treatment [1, 2], but cancer cure is still difficult to achieve except for the early radical surgery. The main reason is, after the above therapies, the presence of residual cancer cells and subsequent tumor recurrence and metastasis, which forms a hindrance for clinical and experimental oncology.

To tackle the residual tumor, the search for efficient necrosis targeting methods may present a breakthrough with great interests. Necrotic tissue, omnipresent in all fast-growing solid tumors [3, 4] and after certain anticancer therapies [5-7], may serve as an anchoring point for a directed tumor therapeutic strategy. Moreover, based on the underlying principle that radiation can be delivered in a targeted way by attaching a certain radionuclide to a molecule or antibody, which then selectively accumulates in tumor necrosis and emits radiation to kill and/or restrain adjacent residual cancer cells. Such a design belongs to internal molecular targeted radiotherapy [8].

Tumor necrosis therapy (TNT) is a promising anticancer strategy, which was first described by Epstein et al [4, 9]. A specific antibody agent ^131^I-chTNT targeting the nuclear antigens in degenerating cells has been applied in clinical treatment. It carries therapeutic radionuclide ^131^I to necrotic areas of solid tumors and exerts cytotoxicities to remnant viable tumor cells [9] to create new necrosis, then subsequent doses of ^131^I-chTNT will be able to extend to these new areas of degeneration, causing a “gangrene-like” effect throughout the tumor so as to therapy. However, so far the clinical therapeutic results appeared unsatisfactory, and the possible explanation is the macromolecule immunogenicity of monoclonal antibodies [10].

Hypericin (4,5,7,4',5',7'-Hexahydroxy-2,2'-dimethylnaphthodianthrone, Hyp), a small molecule compound, was recognized as a prominent necrosis-avid agent [5, 7, 11, 12]. Studies proved radioiodinated Hyp was also found to selectively concentrate in necrotic tissues [13, 14] and sustain a long time [15]. Moreover, ^131^I-Hyp, a prominent necrosis targeting agent carrying therapeutic iodine-131 with high sensitivity and specificity, has been investigated on different types of tumors with preliminary promising outcomes [15, 16].

However, Hyp is labile due to its big conjugate system, especially under light [17]. Besides, with nearly planar conformation, Hyp is poorly soluble and forms nonfluorescent aggregates in aqueous environment [18], which strongly hindered its selective accumulation in necrotic tumor [19]. The coarse aggregates are intercepted by pulmonary capillary network and small ones are cleaned by reticuloendothelial system and substantially retained in the liver and spleen.

**Figure 1:**
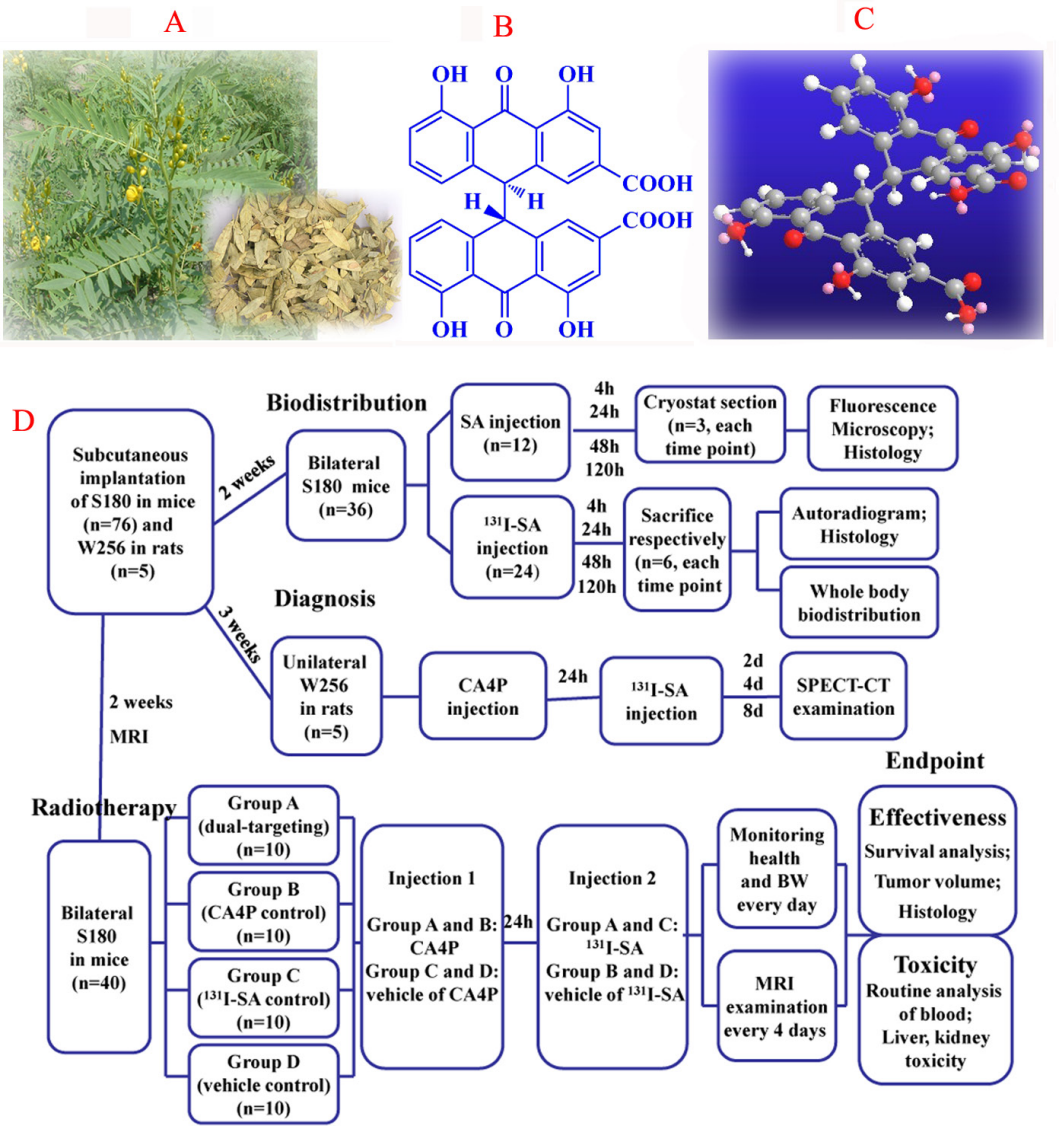
**(A)**, picture of the plant *Senna angustifolia*; **(B)**, two-dimensional structure of SA; **(C)**, three-dimensional structure of SA; **(D)**, flow diagram of experimental procedures (SA, Sennidin A; n, number).

Sennidin A (SA, Figure 1B) and Hyp show the same skeleton with a condensation of two molecules of anthraquinone. However, SA, a kind of median dianthrone derived from the *Cassia* L., *Senna* [20], is free to rotate through a single connecting bond, and spatially arranged in cross with steric hindrance (Figure 1C). This characteristic improves the stability and solubility of the molecule to prevent the formation of aggregations. The necrosis avidity of ^131^I-SA was first investigated in rat models of myocardial infarction with infarct/myocardium distribution ratio of 11.9 [21].

In view of the similar necrosis affinity but better stability and solubility with SA/^131^I-SA relative to Hyp, we hypothesized a combined targeted anticancer strategy, in which a vascular disrupting agent (VDA) [22, 23] first selectively causes massive tumor necrosis that is then targeted by tumoricidal ^131^I-SA, which irradiates the residual cancer cells through constant cross-fire beta particles to achieve local control or cancer cure. Meanwhile, the gamma ray emitted by ^131^I-SA enables scintiscan, hence a theragnostic approach.

To validate this hypothesis, we performed the animal experiments to gain insight into the necrosis avidity or targetability of SA/^131^I-SA by analyzing the biodistribution, in vivo single photon emission computer tomography-computed tomography (SPECT-CT) imaging, autoradiography, and fluoromicroscopy in rodent tumor models. We further investigated the anticancer efficacy and safety of ^131^I-SA as a radiopharmaceutical in combination with a VDA combretastatin A4 phosphate (CA4P) in mice with subcutaneous S180 tumor.

## RESULTS

### Animal models

Bilateral S180 tumor-bearing mice models and unilateral W256 tumor-bearing rat models were established successfully, and they survived the surgery, anesthesia and imaging procedures without any drug administration-related deaths.

### Radiolabeling and in vitro stability

The percentage radioactivity of ^131^I-SA relative to all radioiodine activity was greater than 95% as determined with HPLC, and in vitro stabilities of ^131^I-SA stored in rat serum at 37°C was excellent with RCP over 95% up to 8 days.

### SPECT-CT image

**Figure 2:**
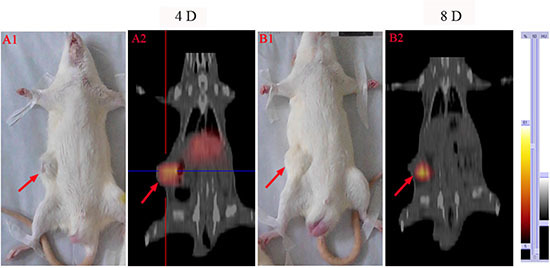
SPECT-CT images at 4 days **(A2)** and 8 days **(B2)** after i.v. injection of ^131^I-SA in W256 tumor-bearing rats. **A1** and **B1**: corresponding gross photographs of unilateral tumor-bearing rats. Red arrows indicate tumor mass.

After SPECT-CT imaging in vivo, unilateral tumors in rats were visible as hotspots from day 2 till 8 after the injection of ^131^I-SA, which became brighter and more focused as a function of time. At day 2 post injection, the whole bodies of rats showed uptakes of ^131^I-SA. However, at day 4, marked tumor uptake of ^131^I-SA and a lower amount of radioactivity in the livers was seen (Figure 2-A2). Up to 8 days (Figure 2-B2), except tumor, all other tissues had no obvious uptakes.

### Whole-body biodistribution of ^131^I-SA

**Figure 3:**
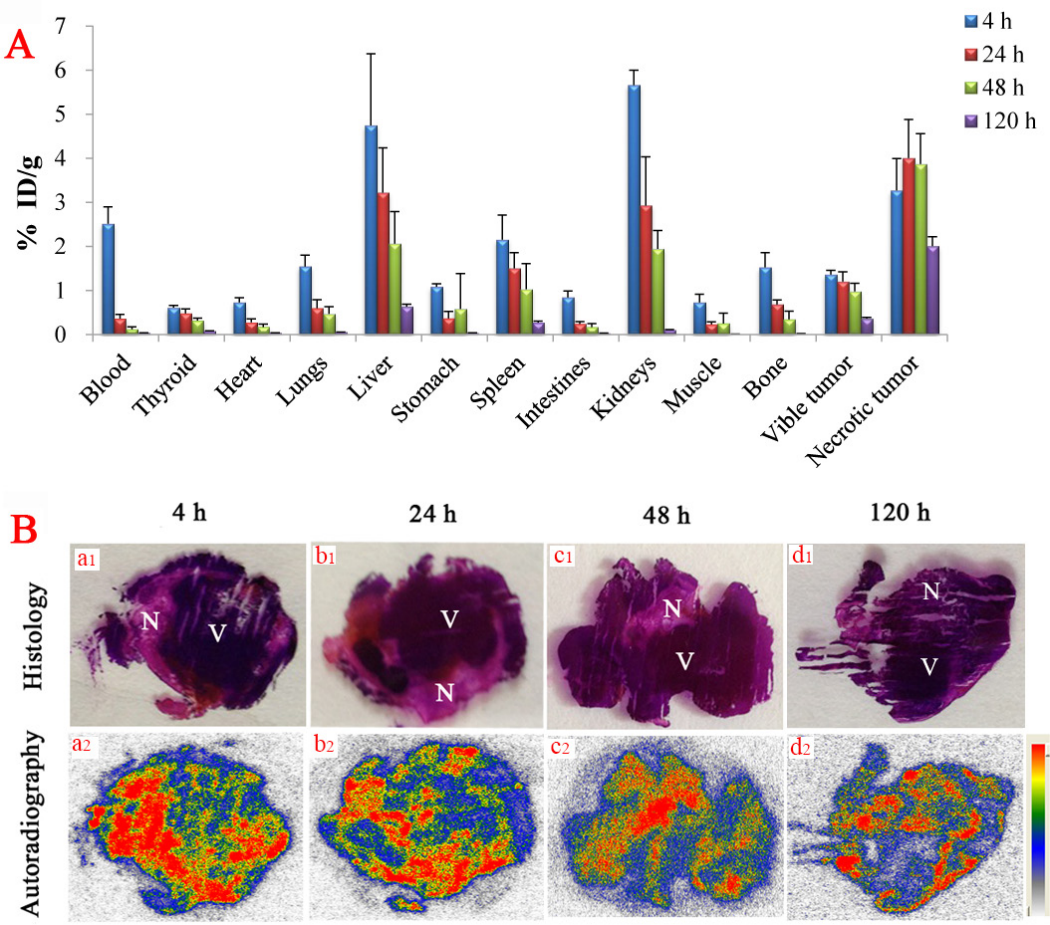
**(A)**, biodistribution of ^131^I-SA studies in bilateral S180 tumor-bearing mice as a function of time (n=6, each time point). Data are expressed as percentage injected dose per gram of tissue (% ID/g). **(B)**, autoradiograph and corresponding contrast-enhanced H&E images of 50-um tumor slices at 4 h (a1, a2), 24 h (b1, b2), 48 h (c1, c2) and 120 h (d1, d2) after i.v. injection of ^131^I-SA (18.5 MBq/kg). N = necrotic area, V = viable tumor area.

Relatively high uptakes of ^131^I-SA were found in the liver and kidney at 4 h post injection, as well as necrotic tumor (Figure 3A). However, except the necrotic tumor, all organs and tissues revealed obvious clearance of radioactivity up to 120 h. The radioactivity quantification of dissected tumor showed high uptake in the necrosis with a necrosis-to-tumor activity ratio of 2.4, 3.2, 4.1 and 7.5 at 4, 24, 48 and 120 h, respectively.

### Autoradiography

Figure 3B represents typical images of the intratumoral distribution of ^131^I-SA at 4 h (Figure 3B-a2), 24 h (Figure 3B-b2), 48 h (Figure 3Bc2) and 120 h (Figure 3B-d2) post injection. At each time point, higher tracer uptake was located mainly in the necrotic tumor regions relative to that in viable tumor, based on the distinction between the necrosis and viable tumor proven by H&E stained slices (Figure 3B-a1, b1,c1 and d1). By a semi-quantitative autoradiography examination, radioactivity ratios of necrosis over liver amounted up to 5.2, 19.8, 23.8 and 31.2 at 4, 24, 48 and 120 h, respectively. The necrotic/viable tumor ratios quantified by autoradiography were higher than by gamma counting. This was due to the fact that viable cells are intermixed with necrotic cells, making separative sampling of necrotic and viable tumor tissues for gamma counting very difficult. Besides, radioactivity inside the necrosis was often inhomogeneous due to relatively poor blood perfusion

### Intratumoral localization of SA

**Figure 4:**
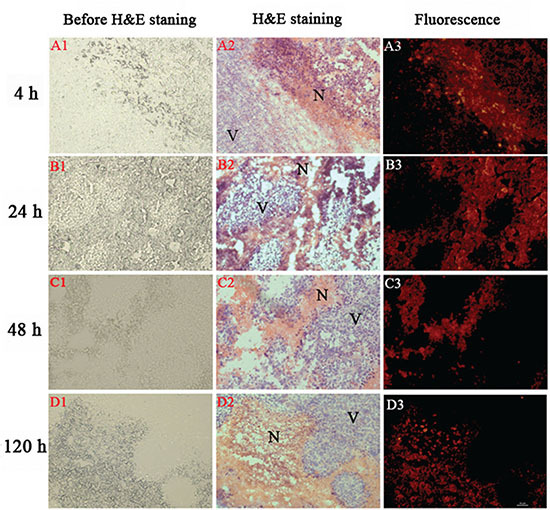
Photomicrography of 5-um tumor sections from S180 tumor-bearing mice as a function of time after i.v. injection of SA (10 mg/kg). Corresponding unstained **(left)**, H&E stained **(middle)** and fluorescence **(right)** pictures: N = necrotic area, V = viable tumor area. Scale bar = 50 um.

To visualize in greater details the selective retention of SA in tumor necrosis over a longer period, intratumoral distribution was analyzed by fluorescence microscopy up to 120 h. Relative to those on the unstained slides (Figure 4-A1, B1, C1, D1), necrotic and viable tumor tissues were distinguished by H&E staining (Figure 4A2, B2, C2, D2), showing different patterns on the microscopic images. Fluoromicroscopic images revealed distinct fluorescence intensity between necrotic and viable tumor at different time points (Figure 4A3, B3, C3, D3), with increasing necrosis-liver ratios of 7.7, 21.6, 30.1 and 42.3 at 4, 24, 48 and 120 h, respectively. At 4 h post injection, the necrotic areas close to the viable tumor showed strong red fluorescence, but the necrotic regions distal to viable tumor showed moderate uptake of SA and viable tumor displayed the lowest uptake (Figure 4A3). Up to 48 h, fluorescence intensity was progressively increasing in necrotic areas, whereas rapidly descending in viable tumor. At 120 h, the fluorescence intensity decreased in tumor necrotic region, but with an increased necrosis to liver ratio. The necrotic-viable tumor ratios quantified by fluorointensity and autoradiography were consistent, which likely endows SA and its radioiodinated analogues with necrosis avidity. This finding suggests that necrosis affinity is a new inherent property of SA, which can be utilized in different fields without or with linking to various isotopes.

### ^131^I-SA mediated targeted radiotherapy in combination with CA4P

### Survival

**Figure 5:**
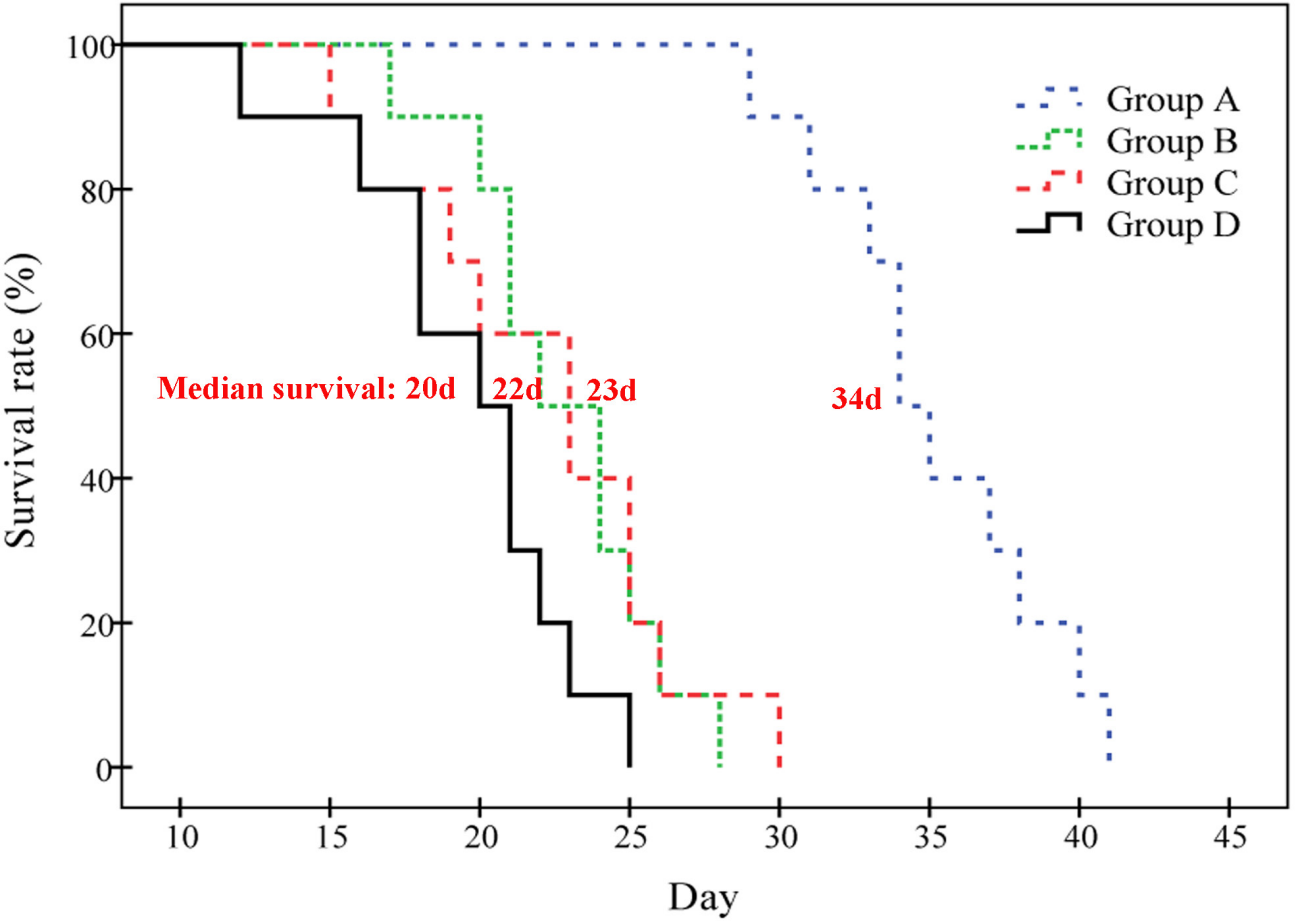
Kaplan-Meier survival curves show the survival probability (%) of the four groups of S180 tumor-bearing mice The median survival was 34 (range 29-41), 22 (range 17-28), 23 (range 15-30) and 20 (range 12-25) days in group A, B, C and D respectively (*P* < 0.01 in group A vs. B, C or D; *P* > 0.05 between group B, C and D).

Only one episode of ^131^I-SA combined with CA4P notably prolonged the survival of tumor-bearing mice, with the median survival of 34, 22, 23 and 20 days in group A, B, C and D, respectively (Figure 5). Significant differences were showed between group A and group B, C or D in survival curves (*P* < 0.001), but no significant difference was found among group B, C and D (*P* > 0.05).

### In vivo MRI

**Figure 6:**
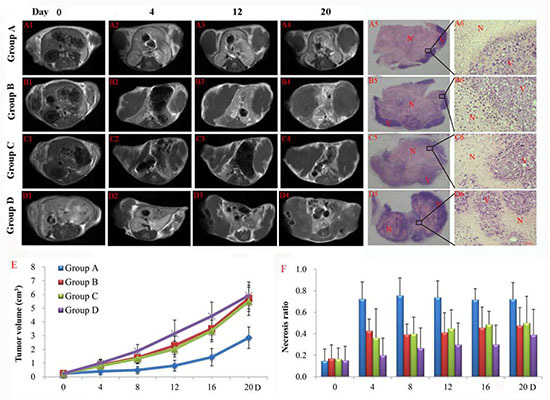
Contrast enhanced T1 (CE-T1) MR images of representative tumor bearing mice from 4 groups on day 0 (A1-D1), 4 (A2-D2), 12 (A3-D3), 20 (A4-D4) Tumors in group B, C and D grew much faster than that of group A. Macroscopic photographs showed extensive and thorough central necrosis surrounded by a thin rim of viable tumor tissues in group A **(A5)**, but presenting a pattern that nests of viable tumor cells are intermixed with foci of spontaneous tumor necrosis in group B **(B5)**, C **(C5)** and especially group D **(D5)** at endpoint. Rectangular frames denote the areas where the photomicrographs **(A6, B6, C6, D6)** were taken which showed the interface between necrotic (N) and viable (V) tumor tissues in 4 groups. Scale bar = 50 um. Tumor growth curve **(E)** and corresponding necrosis ratios **(F)** measured from MRI at baseline, and on day 4, 8, 12, 16 and 20 post-therapy were shown. Significant difference of tumor volume in group A was found compared with that of group B, C and D (*P* < 0.05) from day 8 on. No significant difference was found between group B, C and D (*P* > 0.05) within follow-up of 20 days.

Tumors appeared iso- and hyperintense on T1- and T2-weighted MR images. On CE-T1 images, subcutaneous S180 tumors were enhanced after Gd-DOTA injection, suggesting their hypervascular nature. In group A and B, after injection of CA4P, a non-enhanced central region was surrounded by a thin rim enhancement on CE-T1 images, indicating the presence of massive necrosis and minimum viable tumor (Figure 6A2, B2), but the tumor necrosis region in group A (Figure 6A3) persisted to increase after injection of ^131^I-SA for a longer time compared to group B (Figure 6-B3). In group D, there existed patches of spontaneous necrosis, which were different from the tumors of group A. The borders between necrotic and viable tumor in 4 groups were clearly distinguishable on H&E stained microscopy (Figure 6A6, B6, C6, D6). In group A, exposure of tumor cells to ^131^I-radiation caused marked cell death, characterized by extensive and thorough intratumoral damage (Figure 6A5), but group D presented nests of viable tumor cells intermixed with foci of spontaneous tumor necrosis (Figure 6D5), corresponded to MRI manifestations (Figure 6A2-A4 and D2-D4).

### Tumor volume, tumor doubling time and necrosis ratio

Tumor volumes (Figure 6E) and corresponding necrosis ratios (Figure 6F) were calculated by MRI measurement on different days. At baseline, mean tumor volumes were approximate in group A, B, C and D, respectively (*P* > 0.05). After ^131^I-SA injection, tumor volumes were significantly different between group A and group B, C or D (*P* < 0.05). On day 8, the tumor volumes of group B, C and D were 2.8, 2.7 and 3.8 times as much as that of group A, respectively, with significantly prolonged tumor DT (*P* < 0.01) in group A (13.5 ± 2.1 days) in comparison to group B, C and D (5.6 ± 1.4, 5.4 ± 0.8, 4.4 ± 2.0 days). There was no significant difference (*P* > 0.05) between group B, C and D for tumor volume and DT at each time point.

Spontaneous necrosis existed in S180 tumor, tumor necrosis ratio as measured from CE-T1WI at baseline was about 16% (Figure 6F) in each group. After CA4P injection, a significantly increased necrosis ratio was obtained in group A and B as compared to group C and D (*P* < 0.05). But on day 4, the ratios turned out to be 72 ± 16 % and 43 ± 11 % respectively (*P* = 0.055), indicating a progressively enlarged necrotic region after injection of ^131^I-SA in group A. Significant differences (*P* < 0.05) occurred between group A and group D from day 4 till day 16.

### Tumor weight and volume measurements at endpoint

At endpoint, net weight of tumor was averaged as 5.9 ± 2.0, 6.4 ± 2.8, 6.2 ± 2.1 and 6.8 ± 3.2 g for group A, B, C and D, respectively, corresponding to 7.3 ± 2.9, 7.9 ± 3.1, 7.7 ± 3.2 and 8.5 ± 3.6 cm^3^ of tumor volumes measured with the cylinder method, suggesting a density of about 0.68 g/cm^3^. Tumor weight and volume between four groups had no significant difference (*P* > 0.05) at endpoint. Tumor volumes measured with the cylinder were consistent with that derived from MRI, indicating a reliability of tumor volume measurement and calculation by in vivo MRI.

### Tendency of BW

BW at baseline in group A, B, C and D was 35.7 ± 2.3, 35.6 ± 0.6, 36.1 ± 1.7 and 35.9 ± 1.6 g respectively (*P* > 0.05). Mice in all groups presented a tendency of gaining weight along with tumor growth. On day 15, the BW of group B (44.3 ± 2.1 g), C (45.6 ± 3.7 g) and D (49.5 ± 2.4 g) showed obvious increase, relative to the small change in group A (36.8 ± 1.6 g). However, the mean BW of group A reached 41.8 ± 3.3 g on day 24, which was significantly increased compared with that at baseline (*P* < 0.01).

### Analysis of blood routine

The blood routine indexes of group A, B, C and D before and at two weeks after administration are shown in Table 1. Each of blood indexes showed no significant difference between group A and D (*P* > 0.05). Before and after injection, blood indexes in each group displayed no significant difference (*P* > 0.05). The results revealed ^131^I-SA combined with CA4P made no obvious inhibitory effect on the blood system and immune system after a single dose administration.

**Table 1:** Evaluation of blood routine, and hepatic and renal functions in 4 groups of tumor-bearing mice with different drugs treatment **P < 0.01; *P < 0.5; “a” represents 1 day before CA4P injection; “b” represents 2 weeks after 131I-SA injection; “c” represents 3 weeks after 131I-SA injection. PLT, Platelet; HGB, Hemoglobin; WBC, white blood cell; RBC, red blood cell; ALT, alanine aminotransferase; AST, aspartate aminotransferase; BUN, blood urea nitrogen; CRE, creatinine.

Parameter	Time	Group A	Group B	Group C	Group D
PLT (109*L^−1^)	a	1316 ± 139	1348 ± 135	1301 ± 250	1302 ± 116
	b	1311 ± 102	1275 ± 127	1300 ± 106	1321 ± 102
HGB (g*L^−1^)	a	193 ± 10	192 ± 18	189 ± 18	193 ± 12
	b	189 ± 7	169 ± 17	184 ± 6	182 ± 19
RBC (1012*L^−1^)	a	10.46 ± 0.77	11.24 ± 1.02	10.61 ± 1.75	11.08 ± 0.71
	b	9.65 ± 1.04	9.76 ± 2.03	10.47 ± 0.14	10.05 ± 0.91
WBC (109*L^−1^)	a	11.62 ± 2.36	11.05 ± 1.58	9.30 ± 1.27	12.93 ± 1.53
	b	12.87 ± 2.01	12.44 ± 3.74	10.56 ± 1.04	12.08 ± 3.67
ALT (U/L)	c	117 ± 3**	189 ± 10**	70 ± 3	73 ± 7
AST (U/L)	c	2082 ± 69*	2998 ± 98*	1422 ± 53	1235 ± 138
BUN (mmol/L)	c	13.92 ± 5.77	11.47 ± 2.57	10.02 ± 0.83	10.86 ± 3.46
CER (umol/L)	c	10 ± 2	9 ± 2	10 ± 2	10 ± 1

### Liver and kidney toxicity

Function indexes of liver (AST, ALT) and kidney (BUN, CER) in the serum taken from therapy mice were measured to investigate whether ^131^I-SA may damage liver and kidney due to high uptakes. As shown in Table 1, values of ALT (*P* < 0.01) and AST (*P* < 0.05) in group A and B showed significant difference as compared to group D, respectively. However, liver function indexes revealed no obvious difference between group C and D (*P* > 0.05). This suggests that the raise of liver function indexes in group A and B might be caused by CA4P but not ^131^I-SA. The BUN and CER values displayed no significant difference between group A, B, C and D (P > 0.05). Moreover, histopathologic analysis of liver and kidney revealed no signs of tissue damage.

## DISCUSSION

In this study, SA was efficiently labeled with 131-iodine at a high conjugation rate by Iodogen coating method [24, 25]. It showed prominent targetability with a high sustaining tumor necrosis-liver ratio and quick clearance from non-targeting tissues. Furthermore, ^131^I-SA as a targeted radiopharmaceutical combined with CA4P first demonstrated superb effects for imaging diagnosis and therapy in rodent solid tumor models.

All targeted cancer therapies aim to maximize tumor destruction while minimize side-effects. In this study, quantitative gamma counting of radioactivity in sampled organs revealed a progressive clearance of ^131^I-SA from non-targeting organs such as blood, kidney, but a sustained increasing uptake in tumor necrosis, resulting in a continuous increase in necrosis-to-liver activity ratio over 120 h. Although relatively high radioactivity was shown in liver and kidney at early time, it was eliminated with a short biological half-life via urine and faeces (data unpublished), which minimizes the potential hepatobiliary and renal injuries. Moreover, ^131^I-SA is taken up less in lung and spleen compared with ^131^I-Hyp at 24 h post injection [15], suggesting that SA may overcome the shortcoming with Hyp that, due to poor solubility, forms aggregates cleaned by reticuloendothelial system leading to accumulation in the spleen and lung [5, 13, 24-26]. Besides, ^131^I-SA showed favorable pharmacokinetics with 11.8 h of blood half-life in healthy rats (unpublished data), whereas ^131^I-Hyp displayed a long blood half-life of 30.5 h [27]. SA/^131^I-SA were observed to substantially accumulated in necrotic areas of the tumor at 4 h post injection in our study, whereas ^131^I-Hyp was located mainly in viable tumor tissue and little activity could be detected in necrotic tissue. Only 24 h later, ^131^I-Hyp was transferred to the necrotic region with a time-dependent uptake [14]. With the characteristic early distribution of a high concentration in necrotic areas, ^131^I-SA might be superior to ^131^I-Hyp in the dual targeting therapy in combination with CA4P. Indeed, better accumulation of SA in the necrotic tumor would lower the damage to normal tissues. Besides, SA or its radiolabeled derivatives could be more promising for early imaging diagnosis of myocardial infarction [28, 29].

Early detection of cancer through screening based on imaging is probably the major contributor to a reduction in mortality for certain cancers. Each technique has its own unique applications, advantages, and limitations. Compared with other imaging modalities, PET features high sensitivity and specificity. High tissue radioactivity after administration of ^18^F-FDG corresponds to increased glucose uptake and consumption. Tumor cells are generally metabolically active and will take up more glucose than normal cells. However, PET tends to work better for higher grade tumors and metastasis, as well-differentiated tumors have less metabolic activity. Besides, since inflammation shows as “hot” on a PET scan, it is more difficult to differentiate inflammation from tumors. Therefore, new targeted imaging need to be developed to provide complementary information for improved diagnostic accuracy. In the experiment, we chose CA4P first to cause necrosis in the tumor as the target of ^131^I-SA with high necrosis targetability for sensitively imaging the tumor under treatment. Combining anatomical CT imaging and functional SPECT imaging, we found that substantial ^131^I-SA concentrated in the tumor mass as a hotspot. SPECT-CT images on day 8 showed a very intense accumulation of ^131^I-SA exclusively in the tumor areas, which not only demonstrates the strong diagnostic capacity of ^131^I-SA for detecting tumor over a relatively long period after injection, but also reveals a good stability of radioiodinated SA exerting its persistent targeted radiotherapeutic effect. As clearly shown in this study, the avidity of SA for necrosis offers the opportunity to exploit the compound as a carrier for theragnostic utilities.

As proposed by Blagosklonny MV, tissue-specific targets and combination of potent therapeutic strategies with synergy or selectivity may be effective ways to control tumor [30]. Necrosis tissue appears a perfect tissue-specific target for tumor therapy, which finds a broad tumor spectrum for plenty of indications. In our study, sequential combination of targeted therapies by non-overlapping complementary mechanisms achieved synergetic outcomes with minimal radiation exposure to healthy tissues due to the high target-to-nontarget ratio. Actually, beyond whether drug combination is synergistic, additive or antagonistic, what is essential is that drug combination should be more toxic towards cancer cells compared to normal cells[31].

^131^I-SA has achieved encouraging results in sensitivity and specificity of necrosis targeting by evaluation of biodistribution and in vivo imaging in rodent tumor models. Based on these prominent outcomes, we investigated the potential of ^131^I-SA as a radiotherapeutic agent combined with CA4P, which non-invasively created tumor necrosis. CA4P as a vascular disrupting agent selectively shuts down tumor vasculature and deprives the tumor of blood and oxygen supply, subsequently leads to rapid massive intratumoral necrosis [32, 33], which forms as the next target. Hours after CA4P injection, SA as a small molecular necrosis-avid agent carries and delivers a therapeutic radionuclide iodine-131 to the prior existing or induced necrotic regions in the tumor and kills neighboring residual tumor cells by crossfire radiation.

Therefore, the mean survival time and tumor DT of group A was prolonged significantly (*P* < 0.05) relative to group B, C and D, which indicated an excellent antitumoral outcome of ^131^I-SA to prevent rapid tumor re-growth as seen in group B, C and D. Moreover, the consistent tumor volume inhibition in group A could be monitored by MRI. Neither of the two targeting molecules (CA4P and ^131^I-SA) alone exerted sufficient therapeutic effects to delay tumor growth as evident by group B and C. The possible explanation may be the time coordination and synergistic effect between CA4P and ^131^I-SA administration. CA4P with the dose of 10 mg/kg can kill the tumor from the inside out, leaving layers of remaining viable cancer cells about 20-100 μm of thickness [34], which facilitates the accumulated ^131^I-SA in the necrotic tissue bordering the viable tumor to kill remaining cancer cells. It is noticed that the treatment was not effective late after a single dose of ^131^I-SA in group A. This might be due to the following reasons: 1) the remaining tumor cells repopulate after CA4P therapy; 2) there is no successive radioactivity that can continuously localize into the outstretched new necrotic tissues induced by the single dose of ^131^I-SA; and 3) physical decay of 8 days with radioiodine-131 inside necrosis. It is believed that consecutive ^131^I-SA doses and combination with antiangiogenic agents can be promising in future studies to further improve the anticancer efficacy or realizing tumor eradication.

^131^I-chTNT, a monoclonal antibody based agent with specificity and long retention time in necrotic tumor, has been approved for the treatment of advanced lung cancer in China [35]. In our study, small molecular radiolabeled SA also demonstrated peculiar necrosis avidity by showing high concentration in tumor necrosis with consequent theragnostic effects on bilateral S180 tumor-bearing mice. Moreover, by analysis of blood routine of treated mice, ^131^I-SA did not show myelosuppression, which often happens after ^131^I-chTNT treatment and is an important dose-limiting factor of ^131^I-chTNT [35]. In addition, no adverse effects were detected after ^131^I-SA injection by analysis of hepatic and renal function indexes and histopathologic examination. Comparing to the intratumoral use of ^131^I-chTNT [36], ^131^I-SA may be more advantageous due to its fast diffusion through tumor as a small molecule, and its observed superior in vivo targetability may be attributed to the more favorable pharmacokinetics, i.e. a small–molecular weight (< 1 kDa) necrosis-avid compound versus a macromolecular (> 30 kDa) monoclonal antibody applied for radioimmunotherapy [37].

In conclusion, characteristic of better solubility and stability, lower toxicity and earlier distribution in necrosis, radioiodinated SA shows prominent targetability to necrotic tumor, which allows targeted radiodiagnosis and radiotherapy for residual tumor on top of a prior necrosis-inducing treatment. Thus, with further development and optimization, a new horizon of improved cancer treatability, detectability or even curability can be anticipated.

## MATERIAL AND METHOD

### Animals and tumor models

All the animal experiments and husbandry were approved and supervised by the institutional animal care and research advisory committee. Kunming mice (26–28g) and SD rats (250~300g) were provided by the Experimental Animal Center, Jiangsu Province Academy of Traditional Chinese Medicine, Nanjing, Jiangsu, China. S180 tumor cells (2 × 10^6^) were inoculated subcutaneously to the bilateral flank regions of mice, and W256 tumor cells (2 × 10^6^) were inoculated subcutaneously to the unilateral flank region of rats. Tumor growth was regularly measured and calculated with the formula: tumor volume = (short dimension) ^2^ × (long dimension) × 1/2. Model mice and rats were used when the tumor volume reached 0.3 cm^3^ and 2.5 cm^3^ at 2 and 3 weeks after inoculation, respectively.

### Drug preparation and radiolabeling

SA with a purity >98%, was obtained by hydrolyzing sennoside A, which was separated from *Senna angustifolia* (TengWang pharmaceutical co. Ltd, Haozhou, China, Figure 1A). CA4P (HuaMei technology Co., Ltd, Wuhan, China), was diluted in phosphate buffered saline (PBS) solution (2.5 mg/ml). Sodium iodide (Na^131^I) was supplied by HTA Co., Ltd, Beijing, China. The specific activity was 740 MBq/mL and the radionuclidic purity was > 99%.

The Iodogen coating method was used for radioiodination of SA to form ^131^I-SA. SA was dissolved in dimethylsulfoxide (DMSO) to 2 mg/ml solution. Radioiodination was conducted by adding SA and Na^131^I solutions (volume ratio, 4:1) into Iodogen tube and adjusting pH value with PBS. The mixture was shaken and incubated for 90 min at 45°C, and terminated by removal of reaction mixture. Radiochemical purity (RCP) of ^131^I-SA was determined by HPLC. The ^131^I-SA preparations were formulated in water/polyethylene glycol 400 (PEG 400) mixtures (4:1, v/v).

### In vitro stability

^131^I-SA was incubated in rat serum (volume ratio, 1:20) at 37°C. And then radiolabeling stability was determined by HPLC at the time points of 0.5 h, 12 h, 1d, 2d, 4d, 8d, respectively.

### SPECT-CT imaging on tumor-bearing rats

The thyroid of W256 tumor-bearing rats (n=5) was blocked with Lugol's solution (1.2 g/L) in their drinking water 3 days before and 8 days after the treatment. CA4P was i.v. injected at 10 mg/kg, and 24 h later ^131^I-SA was i.v. injected at 18.5 MBq/kg. A variable-angle dual detector SPECT with 16-slice CT (Symbia T; Siemens Medical Systems, Chicago, IL) was used to scan the rats after ^131^I-SA injection on day 2, 4, 8. Rats were anesthetized (chloral hydrate, 0.3 g/kg) and secured to the head holder of the patient bed in supine position. Anterior and tomographic images were collected using the following acquisition parameters: static image matrix size 128 × 128, acquisition count limit 50000, SPECT tomographic image matrix 64 × 64, and continuous acquisition 15 s/frame × 24 frames.

### Whole-body biodistribution of ^131^I-SA

Twenty-four S180 tumor-bearing mice with their thyroid being blocked by Lugol's solution were received i.v. injection of ^131^I-SA (18.5 MBq/kg) and sacrificed at 4, 24, 48, 120 h (n=6, each time point) post injection. At each time point, necrotic and viable tumor, blood, heart, lung, liver, stomach, spleen, small intestines, kidney, muscle, thyroid gland were sampled. Tissue samples were weighted and radioactivity was measured with an automatic γ-counter (SN-695; Hesuo Rihuan photoelectric instrument, Shanghai, China). The results, corrected for background radiation and physical decay, were expressed as percentage of the injected dose per gram of tissues (% ID/g).

### Autoradiography

Liver and tumor blocks were sampled, imbedded in medium (Tissue-Tek medium, Miles Inc., Elkhart, USA) and frozen in the Cryotome (Shandon FSE, Thermo Fisher Scientific Co., USA). Sections (50 μm) were cut and exposed for 24 h to a high performance storage phosphor screen (Super resolution screen, Canberra-Packare, Ontario, Canada). Using a Phosphor Imager scanner (Cyclone™, Canberra-Packard), the screen was read and the images were processed with Optiquant™ software. Afterwards, the same sections were stained with hematoxylin–eosin (H&E) and digitally photographed. Relative tracer concentration in the necrotic and viable tumor, distinguished based on the histological examination of the H&E stained slices, was estimated by manually drawing regions of interest. Digital light units (DLU) per mm^2^, corrected for background, were then calculated using Optiquant software. Autoradiographic images of liver tissue, showing homogeneous tracer distribution, were taken as a reference.

### Intratumoral localization of SA

Twelve S180 tumor-bearing mice were randomly divided into four groups for assessing intratumoral fluorescence intensity, and sacrificed at 4, 24, 48 and 120 h (n = 3, each time point) after intravenous injection of SA (10 mg/kg). Tumors and liver were excised and cut into cryostat sections (5 um). These slices were examined using a fluorescence microscopy (Axio Primo Vert A1, Carl Zeiss, Gottingen, Germany), and subsequently stained with H&E. With necrotic and viable tumor areas distinguished by H&E-stained slices, relative fluorescence intensity of the necrotic to viable tumor at each time point were obtained by manually drawing ROI using a KS imaging software system (Zen2011 Vision, Carl Zeiss, Hallbergmoos, Germany). Ratios of fluorescence densities in the different regions were then calculated. Fluorescence images of liver slices, showing homogeneous fluorescence distribution, were taken as a reference.

### Combinational therapy protocols

Forty S180 tumor-bearing mice with their thyroid being blocked by Lugol's solution were randomly divided into the following four groups (n=10, each): group A of dual-targeting treatment received i.v. injection of CA4P (10 mg/kg), and 24 h later, ^131^I-SA (185 MBq/kg); group B of CA4P controls received CA4P and solvent of ^131^I-SA; group C of ^131^I-SA controls received ^131^I-SA and solvent of CA4P; group D of dual vehicle controls only received solvents of the two drugs. Their health, activity level, and body weight (BW) were recorded daily. MRI was performed in vivo to monitor and quantify tumor volume and necrosis every four days. Analysis of blood routine was performed with an automatic biochemical analyzer (Modular DP, Roche Co. Germany) before and at two weeks after drug injection, and functional indexes of the liver (alanine aminotransferase and aspartate aminotransferase, ALT and AST) and kidney (blood urea nitrogen and creatinine, BUN and CRE) were examined 3 weeks post injection. At endpoint, animal survival was analyzed, and tumors, liver and kidney were excised for postmortem histopathology verification. Besides, the tumors were weighed and measured using a vernier caliper.

### Magnetic resonance imaging (MRI)

MRI was performed using a clinical 1.5 T whole body MRI scanner (Echo speed, GE Co., USA) with a wrist coil for mouse studies. The mouse was anesthesized with an animal anesthesia machine (Matrx VMR, GENE&I, Beijing, China), and placed supinely in a plastic holder. T1- and T2-weighted spin-echo multi-slice transverse images were acquired, and contrast enhanced T1-weighted (CE-T1W) images were obtained immediately after injection of Gd-DOTA (Dotarem, Guerbet, France) via a caudal vein at 0.2 mmol/kg.

Quantifications of tumor area were measured by manually delineating the outline of the tumor mass on T2-weighted MRI slices. Tumor volume was calculated with the equation: tumor volume = Σ (tumor area on each slice × slice thickness). Tumor doubling time (DT) was calculated based on the formula: DT = (T - T_0_) × log2 / (logV - logV_0_), where (T - T_0_) indicates the time interval between two measurements, V_0_ and V denote the tumor volume at the two points of measurement [38]. On CE-T1 images, the area of central nonenhancing region was delineated to estimate necrosis. The ratios of necrosis were defined as the volume of necrosis over that of entire tumor, i.e. necrosis ratio = Σ (area of necrosis × slice thickness) / (area of whole tumor × slice thickness) × 100%.

### Survival analysis

For survival analysis, the primary endpoint was animal death. Record the animal death every day. Standardized humane endpoint used to euthanize animals was failure to eat and drink for over 3 days and without any limb movement.

The overview of experimental procedures was displayed in Figure 1D.

### Statistical analysis

Statistical analysis was carried out with SPSS for Windows software package (version 19.0; Chicago, IL, USA). Numerical data were recorded as mean ± standard deviation. For other comparisons, a one-way ANOVA was used to test differences among groups, and *P* < 0.05 was considered to be significantly different.

### Disclosure of Potential Conflicts of Interest

No potential conflicts of interest were disclosed by the other authors.
